# Myeloid-Derived Suppressor Cells Promote Metastasis in Breast Cancer After the Stress of Operative Removal of the Primary Cancer

**DOI:** 10.3389/fonc.2019.00855

**Published:** 2019-09-10

**Authors:** Xuelei Ma, Manni Wang, Tao Yin, Yunuo Zhao, Xiawei Wei

**Affiliations:** ^1^State Key Laboratory of Biotherapy, Department of Biotherapy, Cancer Center, West China Hospital, Sichuan University, Chengdu, China; ^2^Lab of Aging Research and Nanotoxicology, State Key Laboratory of Biotherapy, West China Hospital, Sichuan University, Chengdu, China

**Keywords:** MDSC (myeloid-derived suppressor cells), operation, stress, breast cancer, metastasis

## Abstract

**Objective:** To investigate the role of myeloid-derived suppressor cells (MDSC) in cancer progression after the stress of operative removal and the potential treatment value of MDSC depletion.

**Summary Background Data:** Surgery is the most important treatment strategy in breast cancer. Recent research has provided evidence that operations may promote cancer metastases under some circumstances.

**Methods:** A mouse model of breast cancer (administration of the murine breast cancer 4T1 cells subcutaneously) and the stress of operation were used to compare immune responses and survival outcomes. Flow cytometry was performed to detect the expression of CD11b and Gr1 MDSCs in tumor tissues and lung metastases. Cytokine levels were detected with three-color flow cytometry and enzyme-linked immunosorbent assay (ELISA). MDSCs were isolated and co-cultured with 4T1 cells to identify any morphological change with immunofluorescence. The anti Gr-1 antibody was used to detect the function of the anti-Gr1 treatment in breast cancer.

**Results:** The operative stress impaired the overall survival, leading to an increased number of MDSCs that preferentially infiltrated the tumor microenvironment and promoted tumor metastasis. In both *in vitro* and *in vivo* assays, MDSCs induced the epithelial-mesenchymal transition (EMT) of tumor cells through the up-regulation of TGF-beta1, VEGF, and IL-10. Furthermore, a treatment strategy of MDSC depletion was found to reduce pulmonary metastases after operations.

**Conclusions:** The stress of operation could impair the overall survival in mice. The infiltrated MDSCs appear to induce EMT of tumor cells and increase metastases through the up-regulation of TGF-beta1, VEGF, and IL-10 levels. MDSC depletion could be a promising treatment strategy to prevent immune evasion after operations.

## Introduction

Breast cancer is one of the most common malignancies worldwide for women (1). More than 1,380,000 cases of breast cancer are diagnosed each year with over 450,000 deaths related to breast cancer, among which cancer metastases account directly for over 80% of these deaths (2, 3). In recent decades, we have witnessed various means of treating breast cancer, including operative removal, radiotherapy, chemotherapy, and endocrine therapy. In order to control local complications and improve the quality of life, the majority of patients with breast cancer, especially those with large, symptomatic lesions, now receive a comprehensive, surgery-based therapy (4, 5). Several experimental studies, however, have revealed the dual role on operative stress which appears to promote cancer metastasis under certain pathologic conditions (6).

The tumor environment has been reported to play a key role in promoting metastases in patients with solid tumors (7). The operative removal of tumors increases the release of certain cytokines in the tumor environment such as the vascular growth factor, which has been shown to promote cancer metastasis (8). In addition, the procedures of operative removal of the primary tumor may potentially interfere with the immune system. This adverse effect appears to be mediated through inhibition of the immune activities of CD8 T cells and involves a population of cells in the tumor microenvironment known as myeloid-derived suppressor cells (MDSC) (9). MDSCs contribute to the negative regulation of immune responses under many pathologic conditions, including tumor progression (10). Recent work has suggested that MDSCs can directly infiltrate primary tumor tissues, thereby inducing epithelial-mesenchymal transition (EMT) of tumor cells (11), which may be an essential step for tumor cell dissemination and tumor metastasis (12). This process renders cells both migratory and with invasive properties which are closely correlated with patient survival after operation (13).

In this report, we studied the effect of operative stress on the development of metastases after resection of the primary tumor in mice injected subcutaneously with the murine breast cancer 4T1 cells (14). Our results suggested that the stress of the operative procedure impaired the overall survival and activated the response of MDSCs through the up-regulation of TGF-beta1, VEGF, and IL10 levels. We also evaluated the efficacy of early post-operative MDSCs to determine their role in the development of cancer metastasis.

## Methods

### Cell and Cell Culture Conditions

The murine breast cell line (4T1) was obtained from American Type Culture Collection (ATCC, Rockville, MD) and grown in RPMI-1640 Medium supplemented with 10% FBS (Gibco, Eggenstein, Germany), 100 U/mL penicillin (Sigma, Aldrich, USA), and 100 μg/mL streptomycin (Sigma). All cell lines were maintained at 37°C, surrounded by a humidified atmosphere of 5% CO_2_.

### Tumor Tissue Injury Model

All mouse protocols were approved by the Animal Care and Use Committee of Sichuan University (Chengdu, Sichuan, China). To exclude the potential endocrine differences of mixed mouse sexes, 6-week-old female BALB/c mice (HFK Bioscience, Beijing, China) were used to build the breast tumor model. Approximately 1 × 10^6^ 4T1 freshly prepared tumor cells in the exponential stage of growth were injected subcutaneously into BALB/c mice. When the primary tumor diameter reached 4–5 mm (usually day 6 after inoculation), the mice (*n* = 120) were divided randomly into six equal groups as follows: (1) control group; (2) a contralateral skin incision group involving a 15–20 mm long skin incision on the contralateral side from the tumor and symmetrical to where the tumor resection was performed; (3) an ipsilateral skin incision group as the control of operative stress to avoid the impact of skin incision on the primary tumors. The skin incision was 15–20 mm long and 1–3 mm near the primary tumor, without injury to the tumor itself; (4) a 1/4 tumor tissue removal group; (5) a 3/4 tumor tissue removal group; and (6) a whole tumor removal group. Mice in groups 4, 5, and 6 all had a 15–20 mm skin incision first and then 1/4, 3/4, or the entire primary tumors were removed, respectively. Half of the mice of each group (*n* = 10) were used for the survival analysis, while the rest were used for evaluating the number of lung metastases. To ameliorate pain, mice were killed if they exhibited any clinical signs of distress, such as loss of appetite, cachexia, 10% weight loss, loss of mobility, restlessness, respiratory distress, tumor/skin breakdown, or failure to groom.

### Measurement of Lung Metastatic Nodules

After 28 days, the entire lungs and tumor tissues of mice (three mice from each group) were isolated and weighed. Lungs were fixed in 4% paraformaldehyde for counting of lung metastases and measurement of the size (diameters) of metastatic nodules using a dissecting microscope. The total number of nodules and the number of nodules over 3 mm in diameter were also calculated.

### Immunohistochemistry

After 28 days, lung tissues of six groups (three mice from each group) were embedded in Tissue-Tek OCT compound and then frozen in liquid nitrogen. Frozen sections of the primary tumor and lung tissue (all lung cuts of the whole lung tissue, not just the metastases) were used for immunostaining with anti-mouse Gr-1 (Abcam, Cambridge, MA USA) and biotinylated goat anti-rat as the secondary antibody (Abcam, Cambridge, MA, USA). An ABC kit and Diaminobenzidine tetrahydrochloride (DAB) were used as a chromogen to visualize antigens.

### Isolation of Cells From Primary Tumors and Lung Metastases

After 28 days, the primary tumors and lung metastases of the rest of the mice (four mice from each group) were collected, cut into small pieces, and incubated at 37°C for 2 h in 20 ml of RPMI (serum-free) medium containing 1 mg/ml collagenase I (280 U/mg, Gibco) and 2 μl of DNase (2 mg/ml, Sigma). Next, the cell suspension was centrifuged at 300 g for 10 min. The cells were filtered through 40 μm nylon filters and centrifuged at 400 g for 10 min. All living cells were collected from the interface and washed with serum-free RPMI three times.

### Flow Cytometry

Single cells from all six groups were stained with CD11b-PerCP–Cy5.5 or PE, Gr1-FTIC, or PE (BD Biosciences, San Jose, CA, USA) for 30 min at 4°C. CD11b^+^Gr1^+^ MDSCs were stained with antibodies against TGF-beta1, VEGF, and IL-10 (Abcam, Cambridge, MA, USA), with FITC-conjugated secondary antibody labeling for 30 min at 4°C. Then the cells were washed three times with serum-free RPMI before being analyzed. For flow cytometry analysis, cells were acquired with an FACSCalibur flow cytometer (BD Biosciences, San Jose, CA, USA) and analyzed with CellQuest software (BD Biosciences, San Jose, CA, USA) as in previous research (15).

### ELISA

The serum levels of TGF-beta1, IL-10, and VEGF of the mice from the 1/4 tumor removal group and the control group were evaluated on the third day after surgery, using corresponding ELISA kits according to the manufacturers' instructions (eBioscience, ThermoFisher Scientific, Waltham, Massachusetts, USA).

### Prospective Isolation of MDSC (Gr-1 Positive Cell)

Gr-1 positive myeloid cells were isolated by magnetic bead sorting (MACS). For this purpose, myeloid cell suspensions from mice thigh bones were prepared according to a previous method (16). Approximately 1 × 10^8^ cells were added to the buffer. Cells were then incubated with an FcR blocking solution for 10 min at 4°C and A Ly6G biotin antibody for 10 min at 4°C, and cells were enriched using a technique of magnetic-activated cell sorting (magnetic bead sorting), MDSC isolation kit from Miltenyi Biotec, Bergisch Gladbach, Germany. All procedures were performed according to the manufacturer's instructions (17). The purity of isolated cells was determined by standard flow cytometry analysis using a labeled antibody against mouse Gr-1. The purity of isolated Gr-1 positive cells regularly exceeded 95%.

### Co-culture Assays of Epithelial-Mesenchymal Transition (EMT)

4T1 cells were trypsinized, resuspended in culture media, co-cultured with MDSCs, and grew on round cover slips in 24-well plates. Trans-wells were incubated at 37°C containing 1:1, 1:3, 1:5, 1:10, and 1:20 cell number ratio of pre-isolated MDSC cells for 96 h. The morphology of 4T1 cells co-cultured with MDSCs for 24, 48, and 96 h were observed under white light microscope. To explore indirectly the role of MDSCs on EMT of the primary tumor cells, MDSCs were loaded into the upper chambers of a 24 well trans-well insert. After removing non-migrating cells using cotton swabs, inserts were washed in PBS, fixed in 4% PFA (Paraformaldehyde) and prepared for the immunofluorescence assay. Then the cells were permeabilized with 0.1% Triton X-100 in PBS, and stained with mouse monoclonal antibodies directed against E-cadherin and Vimentin (Abcam). The secondary antibodies were Alexa Fluor 488-conjugated donkey-anti-rabbit antibody (Invitrogen, 1:500). DAPI (4',6-diamidino-2-phenylindole) (5 μg/ml) was also stained for 10 min. The images were visualized through a Leica TCS SP5 confocal microscope.

### Model of Post-operative Gr-1 Clearance

Approximately 1 × 10^6^ 4T1 freshly prepared tumor cells at their exponential stage of growth were injected subcutaneously into BALB/c female mice. Then 0.15 mg of anti Gr-1 antibody (BD Biosciences) was injected intraperitoneally at the 7th, 12th, and 18th days after inoculation into mice as per the previous procedure (18). We divided the mice into four groups (10 mice for each group) at the 14th day after inoculation: (1) control group; (2) the operation group with 1/4 tumor tissue removed; (3) the Gr-1 clearance group with intraperitoneal injection of Gr-1 antibody; (4) the combinational treatment group that receives operation and Gr-1 antibody. The tumor tissue removal groups and tumor tissue removal with Gr-1 clearance group received the respective operation on the 14th day after the inoculation. Finally, the lung metastases were analyzed to detect the effect of the anti Gr-1 antibody after 28 days.

### Statistical Analysis

Survival times of mice are recorded as mean ± SEM (standard error of mean), and other data are expressed as the mean ± SD (standard deviation). Statistical significance of the difference as for the number of lung metastases and survival between groups was evaluated with independent-sample Student's *t*-test followed by one-way ANOVA (analysis of variance), using SPSS 17.0. X^2^ test for proportion was used to analyze ELISA and flow cytometry results. Differences were considered significant at *P* < 0.05.

## Results

### Stress of Operative Removal Impairs the Survival and Promotes Lung Metastasis

**Figure 1:**
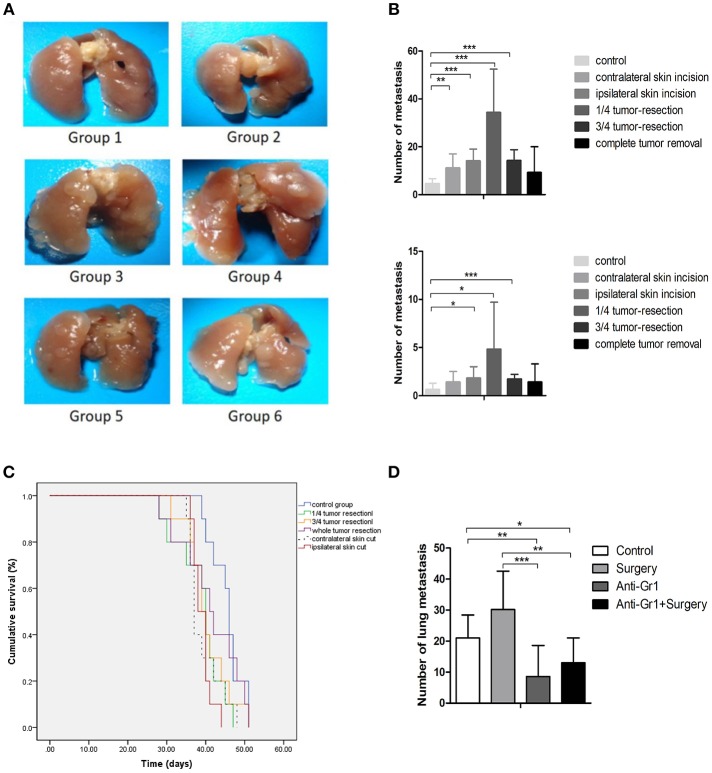
**(A)** Pulmonary metastases of the mice killed on the 28th day after inoculation. Group 1: control group; 2: contralateral skin incision group; 3: ipsilateral skin incision group; 4: 1/4 tumor resection group; 5: 3/4 tumor resection group (resect a large part of the tumor, simulating the effect of subtotal resection on tumor metastasis and tumor microenvironment); 6: whole tumor resection group; **(B)** Quantification of pulmonary metastasis: (i) Total number of metastatic nodules; (ii) The number of metastatic nodules of diameters ≥3 mm; **(C)** The survival outcomes of the six groups (*n* = 10) recorded 2 days after the initial procedure (day 0 is inoculated date); **(D)** The quantification of pulmonary metastases in each group to study the effect of anti-Gr1 treatment on lung metastasis. Mice were divided into four groups: the control group, surgery group (1/4 tumor resection), anti-Gr1 group (The clearance of MDSCs was achieved with anti-Gr1 antibody three times), and anti-Gr1^+^ surgery (1/4 tumor resection) group. Mice went through surgical removal of tumors on the 10th day after inoculation and were sacrificed on the 28th day for the quantification of lung metastasis. ^*^p < 0.05, ^**^p < 0.01, and ^***^p < 0.001.

To investigate the effects of surgical stress on mice in this model of breast cancer, we evaluated the status of pulmonary metastases and the survival of the six groups of mice after different procedures of operative intervention: mice with contralateral skin incision, mice with ipsilateral skin incision, 1/4 tumor resection, 3/4 tumor resection, and whole tumor removal. BALB/c mice were inoculated with 4T1 tumor cells, and primary tumors were removed operatively from mice 10 days later (Figure 1A). The survival outcomes of these 60 mice (10 from each group) were recorded after the initial procedure (day 0 was the inoculation date) and pulmonary metastases of the other 60 mice (10 from each group) were quantified on day 28 according to previously reported method (19).

As indicated in Figure 1B, all groups exhibited tumor metastases during the experimental period. Mice in the control group exhibited a median of 5 ± 2 metastases (ranging from 2 to 8), while the number of metastatic nodules was 11 ± 6 (ranging from 7 to 24) for the contralateral skin incision group, 14 ± 6 (ranging from 8 to 23) for ipsilateral skin incision group, 34 ± 18 (ranging from 12 to 63) for mice with 1/4 tumor removal group, 14 ± 5 metastases (ranging from 10 to 25) for mice with 3/4 tumor removal group, and 9 ± 11 metastases (ranging from 1 to 30) for the group with whole tumor resection. All operational groups showed an increased number of metastatic nodules compared with control (*P* < 0.05). Mice with 1/4 tumor resection exhibited the greatest increase in lung metastases as compared with other groups. Metastatic nodules with diameters of more than 3 mm were considered to be large metastatic nodules. No differences in the incidence of large metastatic nodules were observed between the six groups. The whole tumor resection group failed to demonstrate significantly more metastatic nodules or higher levels of large pulmonary nodules than the control group. Taken together, these results showed that the stress of operation could promote breast cancer metastases. To determine whether primary tumor resection had an effect on survival, we compared the overall survival of mice (*n* = 10). The post-inoculation survival time was 44 ± 2 days for the control group, 39 ± 1 for the contralateral skin incision group, 39 ± 1 for the ipsilateral skin incision group, 40 ± 2 for the 1/4 tumor removal group, 40 ± 2 for the 3/4 tumor removal group, and 42 ± 3 for the whole tumor removal group (Figure 1C).

### Post-operative Depletion of MDSC Decreases Lung Metastasis After Surgery

To systematically verify our hypothesis that MDSCs promote breast cancer metastasis, the anti Gr-1 antibody that specifically targeted Gr1^+^MDSCs in tumor-bearing mice was utilized for the depletion of MDSCs according to previous procedure (18). The anti Gr-1 antibody was used to deplete MDSCs on the 7th, 12th, and 18th days after tumor inoculations. The control group and the Gr-1 clearance group did not receive any operative intervention, whereas the tumor tissue removal group and tumor tissue removal with Gr-1 clearance group received the operations at the 14th day after tumor inoculations. Compared with the control group which developed 21 ± 7 lung metastases, the whole tumor removal group developed 30 ± 12 lung metastases, but failed to demonstrate statistical significance (*P* = 0.059). The Gr-1 clearance groups demonstrated a significant decrease (*P* = 0.006) in the number of metastases, with 9 ± 10 for the anti-Gr1 group and 13 ± 8 for the surgery plus anti-Gr1 group (Figure 1D). These differences in pulmonary metastases suggested that the depletion of MDSCs with the anti Gr-1 antibody reduced post-operative metastasis.

### Stress of Operative Removal Promotes Recruitment of MDSC Into Lung and Tumor Tissues

**Figure 2:**
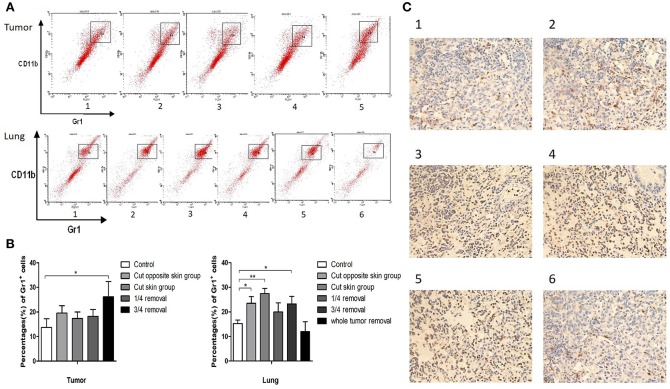
**(A)** Cells in tumor and lung tissues were stained with antibodies against CD11b and Gr1 and CD11b^+^ Gr1^+^ MDSCs were collected for flow cytometry: Group 1: control group; 2: contralateral skin incision group; 3: ipsilateral skin incision group; 4: 1/4 tumor resection group; 5: 3/4 tumor resection group (resect a large part of the tumor, simulating the effect of subtotal resection on tumor metastasis and tumor microenvironment); 6. whole tumor resection group; **(B)** Quantification of CD11b^+^ Gr1^+^ cells by flow cytometry in tumor and lung tissues; **(C)** Gr1 immunohistochemistry staining of the frozen section of mouse lung tissues on day 3 after surgery: (1) control group; (2) contralateral skin incision group; (3) ipsilateral skin incision group; (4) 1/4 tumor resection group; (5) 3/4 tumor resection group; (6) whole tumor resection group. ^*^p < 0.05, ^**^p < 0.01.

To investigate the role of operative procedures on the tumor microenvironment, the primary tumor and lung metastases of 4T1 tumor bearing mice (three mice of each group) were collected for flow cytometry analyses. CD11b^+^Gr1^+^ MDSC cells accounted for 14 ± 4% of cells in the primary tumor tissues of the control group, 20 ± 3% in contralateral skin incision group, 17 ± 3% in ipsilateral skin incision group, 18 ± 3% in 1/4 tumor removal group, and 26 ± 6% in 3/4 tumor removal group. The proportion of CD11b^+^Gr1^+^ MDSC cells in the whole lung tissue were: 15 ± 1% for the control group, 23 ± 3% for the contralateral skin incision group, 27 ± 2% for the ipsilateral skin incision group, 20 ± 4% for the 1/4 tumor removal group, and 23 ± 3% for the 3/4 tumor removal group, and 12 ± 4% for the whole tumor resection group (Figures 2A,B). In addition, we also observed the recruitment of Gr1^+^ cells into lung metastases of post-operation mice with immunohistochemistry (Figure 2C). The 1/4 tumor resection group demonstrated the highest number of metastasis, followed by the 3/4 tumor resection group, ipsilateral skin incision group and contralateral skin incision group. The control group and the whole tumor removal group both exhibited limited visible metastases compared with other groups.

### Recruited MDSC Induce the Epithelial Mesenchymal Transition (EMT) of Tumor Cells *in vitro*

**Figure 3:**
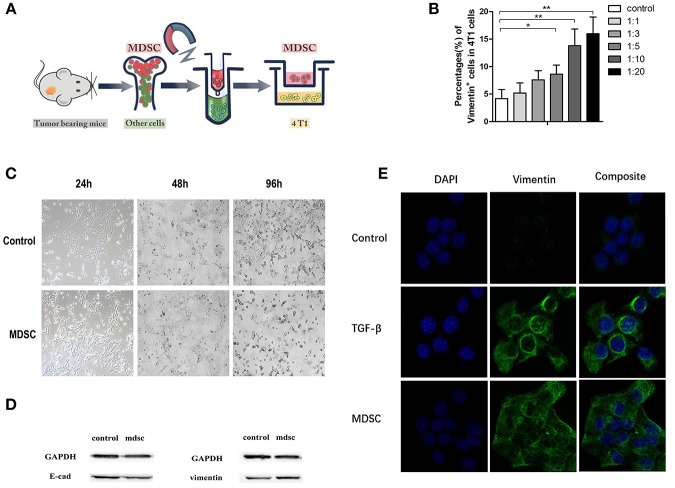
**(A)** Purified MDSCs were extracted with magnetic-activated cell sorting (MACS); **(B)** The quantification of Vimentin-positive subsets in 4T1 cells with the gradient elevation of MDSC concentration in direct co-culture; **(C)**. The morphology of 4T1 cells in control buffer and direct co-culture with MDSCs at the time indicated (24, 48, and 96 h); **(D)** Immunoblots of E-cadherin and vimentin of 4T1 cells in direct co-culture with MDSCs; **(E)** The immunofluorescence of the DAPI/vimentin positive 4T1 cells indirectly co-cultured with control buffer, TGF-βbuffer, and MDSCs. Blue, DAPI (nuclear stain); green, Vimentin (nuclear stain). TGF-beta was added as a positive control for induction of EMT. ^*^p < 0.05, ^**^p < 0.01.

Epithelial Mesenchymal Transition (EMT) theory has been widely acknowledged in explaining tumor metastasis. Purified MDSCs were extracted with magnetic-activated cell sorting (MACS) (Figure 3A). In order to investigate the influence of MDSCs on tumor cell EMT, purified MDSCs (purity of >90%) and tumor cells were co-cultured directly and indirectly. After 96 h of direct co-culture, we noted that with the gradient concentration of MDSCs in the culture, the proportion of Vimentin positive tumor cells increased. Vimentin positive cells accounted for approximately 4 ± 2% of all cells in the control culture, 5 ± 1% in culture with 4T1:MDSC = 1:1, 8 ± 2% in culture with 4T1:MDSC = 1:3, 9 ± 2% in culture with 4T1:MDSC = 1:5, 14 ± 3% in culture with 4T1:MDSC = 1:10, and 14 ± 3% in culture with 4T1:MDSC = 1:20 (Figure 3B). After 24, 48, and 96 h, we observed an obvious change in the shape and structure of 4T1 cells in co-culture with MDSCs compared with those in the control group. Tumor cells gradually grew longer and thinner with their pseudopodia growing until the intercellular junctions disappeared (Figure 3C). Furthermore, tumor cells showed high expression of Vimentin on the cell surface as measured by Western blot assay, while the expression of E-Cadherin decreased markedly (Figure 3D). To explore indirectly the role of MDSCs on EMT of the primary tumor cells, TGF-beta1 was then added to the 4T1 cell cultures as a positive control. EMT could be observed in the culture of the TGF-beta1 group and MDSC group by immunofluorescence (Figure 3E).

### Membrane-Bound Cytokines on MDSCs Appear to Be Critical for EMT *in vivo*

**Figure 4:**
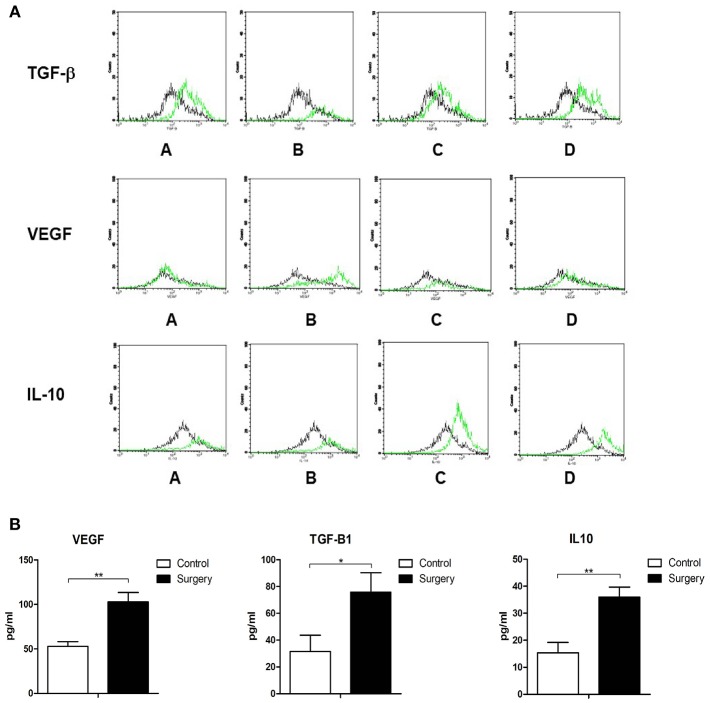
**(A)** MDSCs were extracted from mouse tumor tissues after different operational procedures and analyzed by three-color flow cytometry for the expression of TGF-beta1, VEGF, and IL-10. The control group (black lines) vs. 1/4 tumor resection group (green lines): A: control group vs. contralateral skin incision group; B: control group vs. ipsilateral skin incision group; C: control group vs. 1/4 tumor resection group; D: control group vs. 3/4 tumor resection group; **(B)** The serum expression of TGF-β, VEGF, and IL-10 in the control group and 1/4 tumor resection group on day 3 after operations. ^*^p < 0.05, ^**^p < 0.01.

To further explore the potential mechanisms through which MDSCs affect the primary tumor microenvironment, MDSCs were extracted from mice after different operational procedures and analyzed by three-color flow cytometry for the expression of TGF-beta1, VEGF, and IL-10. TGF-beta1, VEGF, and IL-10 have been shown to be important cytokines inducing EMT. Through an overlay technique, there was an obvious right shift of the TGF-beta1, VEGF, and IL-10 expressions in primary tumors of mice with operations. In other words, the expression of TGF-beta1, VEGF, and IL-10 on CD11b^+^Gr1^+^MDSC was increased in primary tumor tissues (Figure 4A).

### Increased Serum Levels of TGF-Beta 1, VEGF, and IL10

Serum samples of mice exposed to operative stress in the control group and 1/4 tumor resection group were assayed with ELISA to detect the serum levels of TGF-beta1, VEGF, and IL-10. The concentration of these three cytokines was detected (three mice from each group). In the surgery group (1/4 tumor resection), the levels were as follows: VEGF: 102.9 ± 10.5 pg/ml, TGF-beta1: 75.7 ± 14.5 pg/ml, and IL-10: 36.0 ± 3.7 pg/ml; in contrast in control group, VEGF: 52.9 ± 5.3 pg/ml, TGF-beta1: 31.5 ± 12.2 pg/ml, and IL-10: 15.4 ± 3.8 pg/ml (Figure 4B). The serum expression of TGF-beta1, VEGF, and IL-10 increased significantly after the stress of surgery compared with untreated controls.

## Discussion

Breast cancer is one of the most common tumor types and main causes of cancer-related mortality in women worldwide (20). Operative resection of the primary tumor is considered the initial treatment for early-stage, operable breast cancers (21), because the appropriate operation helps remove areas of necrotic tumors that are inaccessible to therapeutic drugs (22) and increases the efficacy of adjuvant treatments in cancer patients. Other positive effects of primary tumor resection include the restoration of the patients' immune system, improvement of the patient's nutritional status, and the control of further metastatic spread (23). Our study was designed to explore these differences and to shed light on the role of surgical stress on metastases in mice with breast cancer. The results suggest that the stress of operative removal changes the microenvironment in the primary tumor mass and the areas of lung metastases, which might be the one key determinant of patient survival and distant metastases.

**Figure 5:**
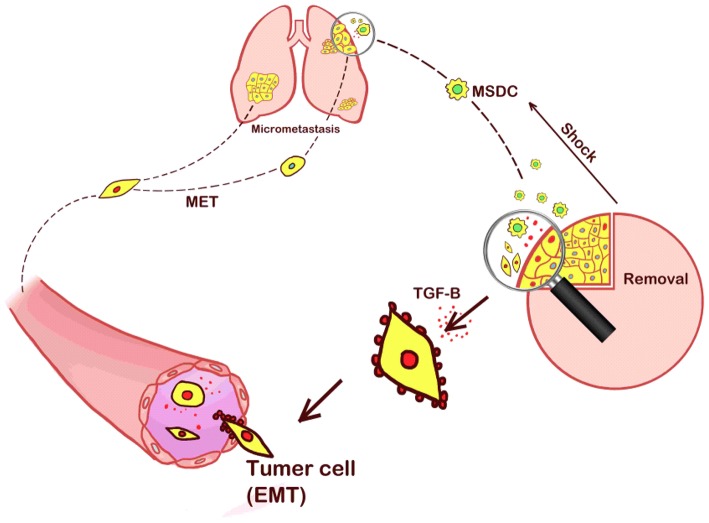
Our hypothesis that the increased lung metastases were caused by the stress of the operative procedure that recruited MDSCs to the primary tumor sites. MDSCs promoted EMT and the release of tumor cells into the circulation, thereby increasing the presence of distant metastasis.

On the other hand, some researchers have reported the negative effects of surgical stress on the growth kinetics of metastases (24). Operative resection of the primary tumor decreases the inhibition of tumor-induced angiogenesis, potentially leading to increased growth of metastases (25, 26). As early as the 1910s, studies have reported that implanted tumors rarely developed spontaneous metastases, unless the primary implant was incompletely removed (27). The results were further detailed by a study that incomplete primary tumor resection could lead to larger metastases in mice than in control groups (28). Similar findings were later reported that significant increases in the number of micro-metastases and total lung weight were detected in mice 13 days after surgical removal of primary tumors (29). Moreover, earlier work by Dr. Folkman and his team suggested that the removal of a primary tumor in mice could lead to the exponential growth of its lung metastases (30), and that before resection, the presence of a primary tumor was correlated with an increased level of angiostatin. The operative stress was associated with worse survival in the mouse model, considering tumor volume might be an important factor (14, 26). In the clinical setting, previous reports identified no better outcome in infants undergoing primary tumor resection than those without surgical treatment (31). In our experiment, we hypothesized that the increased lung metastases were caused by the stress of the operative procedure that recruited MDSCs to the primary tumor. MDSCs promoted EMT and the release of tumor cells into the circulation, thereby increasing the presence of distant metastasis. Our hypothesis is presented in Figure 5.

The microenvironment related to the tumor itself is believed to represent a major impediment to the therapeutic effectiveness of many cancer treatments (32). MDSCs are immature myeloid cells that can act as immunosuppressive agents and are increased in patients with malignancies, as well as in mouse tumor models (33). It has been reported that the increase of MDSCs in the spleen could be prevented by primary tumor resection (34). The suppression of antitumor immunity by MDSCs has been attributed to various mechanisms, including interference with T cell activation and the development of dendritic cells. Different operative procedures can affect T cell function in the tumor microenvironment (35) by directly disrupting the function of CD4^+^ and CD8^+^ T cells (10, 36). In this regard, MDSCs can inhibit immune responses and at the same time promote immune escape of tumor cells leading to metastases (37, 38). Several studies have suggested that inhibition of the surgery-induced dysfunction of NK cells can help to prevent postoperative metastasis (39, 40). MDSCs appear to be able to suppress innate immunity through down-regulation of NK cell function (41), possibly by inhibiting NK cell cytotoxicity, NKG2D expression, and interferon production through membrane-bound TGF beta1 (41). In this mouse model of breast cancer, we found that MDSCs were rapidly recruited to the site of the primary tumors, suggesting that the acute stress after surgery was associated with the release of MDSCs rather quickly in the postoperative period. Our results supported similar findings with previous literature that the increased populations of MDSCs were related to the development of lung metastasis (42, 43). However, the exact association between MDSCs and increased tumor cell metastases still remains unclear. One potentially important finding was that the TGF-beta family could lead to EMT in the primary tumor which increased the metastasis by promoting MDSC infiltration into the tumor microenvironment (44). EMT appears to be activated during cancer invasion and metastasis. This biologic process allows a polarized epithelial cell, which normally interacts with the basement membrane via its basal surface, to undergo multiple biochemical changes that enable it to assume the phenotype of a mesenchymal cell. In our *in vitro* study, we found that the EMT of tumor cells was induced and that the portion of cells undergoing EMT increased with increasing proportion of MDSCs. Previous reports also confirmed that the up-regulation of TGF-beta family genes could mediate EMT (45–47).

In the *in vivo* arm of our study, we detected increased tissue and serum levels of TGF-beta 1 in mice with lung metastases after the different operative procedures. TGF-beta can be produced by MDSCs as a suppressive cytokine that inhibits antitumor immune responses (41), which in turn promotes tumor progression by recruiting MDSCs to the tumor microenvironment (44). The up-regulated expression and serum levels of TGF-beta have been reported to indicate the advanced stage, metastasis, and worse prognosis in cancers such as lung cancer (48). We also detected the increased proportion of MDSC subgroups with high expressions of membrane-bound TGF-beta 1, VEGF, and IL-10, both in residual tumor tissues and in lung metastases after operations. A previous study suggested that VEGF contributed to tumor progression, which was positively correlated with MDSC levels in cancer patients (49). The upregulation of VEGF on MDSCs of primary tumors could in turn promote the activation of the MDSCs and therefore, might be a potentially useful target to manipulate MDSC expansion (10). Angiogenesis is not only a key stimulator for the growth of the primary tumor, but also important for the expansion of established metastases. Angiostatin, a plasminogen fragment characterized with antiangiogenic effects, was able to suppress metastasis growth *in vivo* by inhibiting tumor angiogenesis (30). They also found that as early as 5 days after primary tumor resection, lung metastases were soon infiltrated with endothelial cells, indicating that tumor metastases were associated with the formation of blood vessels (29). IL-10 can be expressed by MDSCs as a stimulator for tumor progression (50, 51). High serum levels of IL-10 have previously been detected in patients with ovarian cancer (52–54), and are consistently associated with advanced progression and poor prognosis in multiple cancer types (55–57). A more recent study identified the CD11b^+^CD11c^+^ MDSC as an important IL-10-producer in tumor microenvironments which helps to establish a friendly environment for tumor growth (58). Moreover, selectively blocking the IL-10 signaling is an effective therapeutic strategy which potently inhibits MDSC activities (58). These results suggested that operations could promote the recruitment of MDSCs to residual primary tumors as well as lung metastases, and is associated with increased expression and release of cytokines such as TGF-beta 1, VEGF, and IL-10 by MDSCs. Those cytokines, together with increased MDSCs, can be a possible explanation for the increased metastases after operations.

MDSCs have been considered as potential effectors of cancer immune evasion and can enhance tumor-induced immune suppression in the tumor microenvironment (59, 60), making it a novel target for therapeutic intervention. Some recent work has suggested the potential of MDSC depletion as a strategy for cancer treatment (61). Targeted depletion of MDSCs in pancreatic cancer increases the intra-tumor accumulation of adaptive immunity and remodels the tumor microenvironment (62). Anti Gr1 antibody is widely used for depletion of MDSCs (63). In a previous study, we found that si-A20 expression or anti Gr1 antibody treatment could induce MDSC apoptosis and exert an anti-tumor effect (18). The present findings suggest that the depletion of MDSCs by the anti Gr1 antibody decreases post-operative lung metastases. And in the future, the adverse effects of recruited MDSCs, possibly before and also in selected situations after surgery, can be eliminated by the anti Gr1 antibody.

## Conclusions

The stress of operative procedures promotes MDSC recruitment into the residual primary tumor and lung metastasis. Membrane-bound TGF-beta 1, VEGF, and IL-10 on MDSCs could induce tumor cells to undergo EMT leading to tumor cell metastases. The MDSC depletion with the anti Gr1 antibody both before as well as at the time of resection of primary tumors provides a promising approach for the prevention of metastases of breast cancer.

## Data Availability

All data generated or analyzed during this study are included in this article.

## Ethics Statement

All mouse protocols were approved by the Animal Care and Use Committee of Sichuan University (Chengdu, Sichuan, China).

## Author Contributions

MW collected, analyzed the data, and wrote the article. XM and XW provided the idea. TY modified the article. YZ edited the pictures. All authors reviewed the manuscript.

### Conflict of Interest Statement

The authors declare that the research was conducted in the absence of any commercial or financial relationships that could be construed as a potential conflict of interest.
